# Provably secure identity-based identification and signature schemes from code assumptions

**DOI:** 10.1371/journal.pone.0182894

**Published:** 2017-08-15

**Authors:** Bo Song, Yiming Zhao

**Affiliations:** Laboratory of Cryptography and Information Security, Software School, Fudan University, Shanghai, China; University of Texas at San Antonio, UNITED STATES

## Abstract

Code-based cryptography is one of few alternatives supposed to be secure in a post-quantum world. Meanwhile, identity-based identification and signature (IBI/IBS) schemes are two of the most fundamental cryptographic primitives, so several code-based IBI/IBS schemes have been proposed. However, with increasingly profound researches on coding theory, the security reduction and efficiency of such schemes have been invalidated and challenged. In this paper, we construct provably secure IBI/IBS schemes from code assumptions against impersonation under active and concurrent attacks through a provably secure code-based signature technique proposed by Preetha, Vasant and Rangan (PVR signature), and a security enhancement Or-proof technique. We also present the parallel-PVR technique to decrease parameter values while maintaining the standard security level. Compared to other code-based IBI/IBS schemes, our schemes achieve not only preferable public parameter size, private key size, communication cost and signature length due to better parameter choices, but also provably secure.

## 1 Introduction

In 1994, Shor published a quantum algorithm [1], which could ruin public key cryptography based information security as we know it today. With the development of quantum computers, NIST (National Institute of Standards and Technology) made a call for quantum resistant algorithms in 2016. Code-based cryptography represents one of few such alternatives supposed to be secure in the post-quantum world. McEliece [2] proposed first code-based public cryptosystem in 1978. Since then, a wide range of code-based cryptographic primitives has been proposed, such as digital signatures, identification protocols and hash functions [3]. Moreover, compared to traditional cryptosystems, many of them also show the advantage on fast computation [3, 4].

At the same time, public key management is one of the most critical issues on multi-party communications and public key cryptography. In 1984, Shamir [5] introduced identity-based public key cryptography. The key point is that the public key of a user can be his identity *id*, i.e., public information about that user, such as a name, a phone number, or an e-mail address. The motivation behind identity-based systems is to largely simplify the management of public keys for the authentication of users. In such systems:

1. Knowledge of a name or emails alone suffices for cryptographic operations such as verification of a digital signature.
2. No need for a public directory, i.e., a database containing public keys or certificates.
3. Trusted authority is needed only during a set-up phase.

Therefore, it is very appealing to make fundamental cryptographic primitives, i.e., identification protocol and digital signature, gain such advantages [6, 7] for more practical applications.

Cryptographic identification protocol [6, 8] is designed to eliminate the security and privacy issues in traditional identification. For traditional identification, the server checks whether the submitted secret key is identical to the key stored in the database. However, there are increasing concerns on the security of such user-selected passwords, secret key leaking, and the attacks on such databases. In contrast, the identification protocol is a zero-knowledge protocol, so the verifier or the server only knows the public key (or just identity in identity-based systems) of the prover or the user. Through a challenge-response manner, it checks the validity of the prover.

Meanwhile, the digital signature is a well-known cryptographic tool for demonstrating the authenticity of digital messages or documents. When it comes to identity-based digital signature, the verifier only needs to know the name or email address instead of a long and awkward public key of the signer.

In 2009, Cayrel et al. [8] proposed state-of-the-art identity-based identification (IBI) and signature (IBS) schemes from code assumptions, or the mCFS-Stern scheme. It can be regarded as a combination of the CFS signature scheme [9] and the Stern identification protocol [10, 11]. There are several improved mCFS-Stern schemes are proposed since then. Alaoui et al. [12] uses quasi-dyadic Goppa codes in the user key extraction algorithm to reduce public key size. Cayrel et al. [13] proposes a way to modify the Stern protocol with the q-ary syndrome decoding problem so that the cheating probability of each round reduced from $\frac{2}{3}$ to $\frac{1}{2}$, and thus reducing the communication cost and signature length. Aguilar et al. [14] adapt such technique with double circulant codes to optimze mCFS-Stern protocol.

However, with the development of code-based cryptography, security and efficiency issues on the mCFS-Stern scheme have arisen. Firstly, Faugère et al. [15] developed a high rate distinguisher for Goppa codes so that the security proof of mCFS-Stern scheme is invalidated. Secondly, Bleichenbacher [16] showed an attack based on the Generalized Birthday Algorithm [17]. It decreases the security level from $2^{\frac{mt}{2}}$ to $2^{\frac{mt}{3}}$ so that increased parameters are required to maintain a required security level, i.e., 2^80^. Thirdly, other improved mCFS-Stern schemes, either using quasi-dyadic Goppa codes [12] or modifying the Stern protocol [13, 14], are vulnerable to the very recent structural attack on quasi-cyclic (QC) or quasi-dyadic (QD) alternant/Goppa codes [18] and could be broken in less than two minutes.

### Our contribution

In this paper, we first propose provably secure identity-based identification and signature schemes with the PVR signature [19] technique applied in the user key extraction algorithm. It does not rely on the indistinguishability between a binary Goppa code and a random code, whereas it is required in the CFS signature scheme and has been invalidated by the distinguisher. Moreover, we present the parallel-PVR technique, inspired by the parallel-CFS technique [20]. It decreases the value of parameters while maintaining the standard security level, which used to be highly influenced by the Bleichenbacher attack. It also might be of an independent interest in the code-based digital signature. Finally, we adapt the Or-proof technique [7, 21] to our schemes so that they are secure against impersonation under active and concurrent attacks (id-imp-ca) instead of passive attacks (id-imp-pa). Currently, our schemes are the only code-based IBI/IBS schemes which are provably secure and they also achieve better efficiency compared to the mCFS-Stern scheme.

### Organization

The paper is organized as follows: In Section 2, we provide some preliminaries. We propose basic provably secure IBI/IBS schemes from code assumptions in Section 3. In Section 4, we further optimize our schemes with parallel-PVR and improve their security level. We discuss the parameters in Section 5 and conclude in Section 6.

## 2 Preliminaries

We first provide some backgrounds and notions for code-based cryptography and then review the definition of identity-based identification and signature schemes in this section.

### 2.1 Code-based cryptography

Let *C* denotes a binary linear-error correcting code of length *n* = 2^*m*^ and dimension *k*, or a [*n*, *k*] code is a subspace of dimension *k* of $F_{2}^{n}$. The elements of the set *C* are called *codewords*. A generator matrix *G* of a [*n*, *k*] code *C* is a matrix whose rows form a basis of *C*. A *parity check matrix* *H* of *C* is an (*n* − *k*) × *n* matrix whose rows form a basis of the orthogonal complement of *C*. The *syndrome* of a vector $x\in F_{2}^{n}$ with respect to *H* is the vector $Hx^{T}\in F_{2}^{n-k}$. The error correcting capability of the code is $t⩽[\frac{d-1}{2}]$, where *d* is the minimum Hamming distance of *C*. The Hamming distance between two words refers to the number of coordinates where they differ. The Hamming weight of a vector *x*, or wt(*x*), is the number of non-zero entries. We use the symbol $\overset{\$}{\leftarrow }$ to denote the uniformly random selection, and use the symbol ‖ to denote the concatenation.

#### 2.1.1 The Bounded Decoding problem (BD)

Let *n* and *k* be two positive integers and *n* ≥ *k*.

**Input.** $s\overset{\$}{\leftarrow }F_{2}^{n-k}$, $\omega =\frac{n-k}{\log_{2}n}$, and $H\overset{\$}{\leftarrow }F_{2}^{(n-k)\times n}$.
**Find.** a word $x\in F_{2}^{n}$ such that wt(*x*) ≤*ω* and *Hx*^*T*^ = *s*.

The BD problem is showed to be NP-complete in [22]. The advantage of a probabilistic polynomial-time (PPT) algorithm solving the BD problem for [*n*, *k*] code should be negligible.

#### 2.1.2 Randomized courtois-finiasz-sendrier signature scheme

Courtois et al. [9] first proposed a practical code-based signature scheme, or the *CFS* scheme. Dallot [23] proposed a randomized variant *mCFS* and proved mCFS is strongly unforgeable under chosen message attack at that time. The scheme works as follows:

**Key Generation.**
  Set $t=\frac{n-k}{\log_{2}n}$. The private key is a (*n* − *k*) × *n* parity check matrix *H* of a *t*-error correcting Goppa code, a non-singular matrix *Q* and a permutation matrix *P*. The public key is the (*n* − *k*) × *n* matrix $\tilde{H}=QHP$.
**Sign.**
  1. $i\overset{\$}{\leftarrow }F_{2}^{n-k}$
  2. Use the decoding algorithm to decode *Q*^−1^*h*(*m*‖*i*). h is a cryptographic hash function and m is the signing message.
  3. If the decoding result *x*′ = ⊥, go back to step 1. It needs *t*! decodings on average.
  4. Output (*i*, *x* = *x*′ *P*).
**Verify.**
  1. Compute $s^{\prime }=\tilde{H}x^{T}$ and *s* = *h*(*m*‖*i*).
  2. If *s*′ = *s* and wt(*x*) ≤ *t*, then the signature is valid; otherwise return false.

The security reduction of the scheme relies on the indistinguishability between a binary Goppa code and a random code. However, it is invalidated by a high rate distinguisher for Goppa codes [15]. Recently, Mathew et al. [19] proposed the PVR signature scheme. which altered the key-construct of the CFS signature and presented a formal proof of PVR without such assumption. Meanwhile, Bleichenbacher [16] showed an attack so that it has to increase the parameters of CFS such as *m* and *t* to achieve the same security level. Finiasz proposed the Parallel-CFS [20], which resisted such attack through performing multiple complete-decoding-based signing processes.

#### 2.1.3 The stern identification scheme

Stern [10, 11] proposed a standard identification scheme based on error-correcting codes. Given a random public (*n* − *k*) × *n* matrix *H* over $F_{2}$. Each user *P* receives a secret key *x* of *n* bits and wt(*x*) = *t*. The public key of *P* is *s* = *Hx*^*T*^. To prove to a verifier *V* that the prover *P* is the user corresponding to the public key *s*, *P* runs the following identification protocol with his secret key *x*:

**Commitment.**
  *P* randomly chooses $y\in F_{2}^{n}$ and a permutation *σ* of {1, 2, ⋯, *n*}. *P* sends to *V* the commitments *c*_1_, *c*_2_, and *c*_3_ such that:
  *c*_1_ = *h*(*σ*‖*Hy*^*T*^);*c*_2_ = *h*(*σ*(*y*));*c*_3_ = *h*(*σ*(*y* ⊕ *x*)), where *h* denotes a cryptographic hash function.
**Challenge.**
  *V* randomly sends *b* ∈ {0, 1, 2} to *P*.
**Answer.**
  If *b* = 0: *P* reveals *y* and *σ*.
  If *b* = 1: *P* reveals (*y* ⊕ *x*) and *σ*.
  If *b* = 2: *P* reveals *σ*(*y*) and *σ*(*x*).
**Verification.**
  If *b* = 0: *V* verifies that *c*_1_, *c*_2_ have been honestly calculated.
  If *b* = 1: *V* verifies that *c*_1_, *c*_3_ have been honestly calculated.
  If *b* = 2: *V* verifies that *c*_2_, *c*_3_ have been honestly calculated, and wt(*σ*(*x*)) is *t*.
**Repeat.**
  Repeat the above four steps for *γ* times so that the expected security level is reached.

**Remark.** *During the verification step, if* *b* *equals 1*, *Hy*^*T*^ *can be directly derived from* *H*(*y* ⊕ *x*)^*T*^ *through:* *Hy*^*T*^ = *H*(*y* ⊕ *x*)^*T*^ ⊕ *Hx*^*T*^ = *H*(*y* ⊕ *x*)^*T*^ ⊕ *s*.

**Theorem 1.** *The Stern identification protocol* (*P*, *V*) *is a proof of knowledge system with knowledge error* ${\left(\frac{2}{3}\right)}^{\gamma }$ [11].

### 2.2 Identity-based identification and signature

In this section, we review the definition and security model for an identity-based identification scheme (IBI) following [6, 21]. An identity-based signature scheme (IBS) can be derived from IBI through Fiat-Shamir heuristic [24].

#### 2.2.1 IBI definition

An identity-based identification scheme IBI = (MKGen, UKGen, $\bar{\text{P}}$, $\bar{V}$) consists of four PPT algorithms as follows:

**Master key generation algorithm (MKGen).**
  It takes 1^*κ*^ as input, where *κ* is the security parameter. It returns a pair of the system public parameters *mpk*, and the master secret key *msk*, which is known only to a master entity.
**User key extraction algorithm (UKGen).**
  It takes *msk* and an identity *id* ∈ {0, 1}* as inputs. It returns a user secret key *usk*[*id*].
**Interactive identification protocol** ($\bar{P}$, $\bar{V}$).
  The prover *P* with identity *id* runs algorithm $\bar{P}$ with initial state *usk*[*id*], and the verifier *V* runs $\bar{V}$ with (*mpk*, *id*). When $\bar{V}$ returns ‘accept’ or ‘reject’, the protocol ends.

*Completeness:* For all $\kappa \in N,\;id\in {\left\{0,1\right\}}^{*},\;\left(mpk,msk\right)\leftarrow$ MKGen(1^*κ*^), and *usk*[*id*]← UKGen(*msk*, *i*), the protocol between $\bar{P}$ with initial state *usk*[*id*] and $\bar{V}$ with (*mpk*, *id*) always ends with $\bar{V}$ outputing ‘accept’.

#### 2.2.2 Security models

There are three security models, i.e., impersonation under passive (id-imp-pa) attacks, active (id-imp-aa), and concurrent (id-imp-ca) attacks. The id-imp-pa secure implies the adversary can query the conversation between *P* and *V* while the id-imp-aa/ca secure implies the adversary acts a malicious *V* to communicate with *P*. The id-imp-ca security implies the adversary can concurrently issue proving queries instead of only one interactive query at a time for the id-imp-aa secure. The formal definitions are shown below:

An IBI scheme is said to be id-imp-atk secure where atk = pa/aa/ca if any adversary A has a negligible advantage in the following game with a simulator S:

**Setup.**
  S takes a security parameter *κ*, generates (*mpk*, *msk*) ← MKGen(1^*κ*^), and gives *mpk* to A. S initializes three empty user sets: *HU*, *CU*, and *PS*, which stand for honest users, corrupted users, and provers’ sessions respectively.
**Phase 1.**
  A adaptively issues following queries:
  **Initialization query (*id*).**
    If *id* ∈ *HU* ∪ *CU*, return ⊥. Otherwise, run *usk*[*id*] ← UKGen(*msk*, *id*), add *id* into *HU*, and return whether the above process is successful.
  **Corruption query (*id*).**
    If *id* ∉ *HU*, return ⊥. Otherwise, remove *id* from *HU*, add it into *CU*, and return *usk*[*id*].
  **Conversation query (*id*). (atk = pa)**
    If *id* ∉ *HU*, return ⊥. Otherwise, return a transcript of a transaction between *P* with *usk*[*id*] and *V* with *mpk* and *id*.
  **Proving query (*id*, *s*, *M*_*in*_). (atk = aa/ca)**
    If *id* ∉ *HU*, return ⊥. If (*id*, *s*) ∉ *PS*, then adds (*id*, *s*) to *PS* where *s* is a session index. If atk = aa, there should be only a single session at any one time. If atk = ca, A could maintain several sessions concurrently. It picks a random bit *τ*, and sets a state of the prover *st*_*P*_[(*id*, *s*)] ← (*mpk*, *usk*[*id*], *τ*). It acts as *V* to obtains (*M*_*out*_, *st*_*P*_[(*id*, *s*)]) from *P* with (*M*_*in*_) and *st*_*P*_[(*id*, *s*)], where *M*_*in*_ and *M*_*out*_ are communication messages between *P* and *V*. Return *M*_*out*_.
**Challenge.**
  A outputs a target identity id* ∈ *HU*, and S removes id* from *HU* to *CU*.
**Phase 2.**
  Same as Phase 1.
**Condition.**
  A wins the game if S halts with *V* outputting ‘accept’. The advantage is defined as ${\text{Adv}}_{A}^{\text{id}-\text{imp}-\text{pa}}\left(\kappa \right)$ = Pr[*V* outputs ‘accept’].

#### 2.2.3 Code-based IBI schemes

Cayrel et al. [8] proposed the first IBI/IBS scheme from code assumption with security proof. It combines the mCFS signature scheme and the Stern identification protocol (mCFS-Stern) as follows:

**MKGen.**
  Set *mpk* and *msk* as the public parameters and the private key of mCFS scheme respectively.
**UKGen.**
  Generate a mCFS signature (*i*, *x*) of the identity *id*. Set *usk*[*id*] = (*i*, *x*).
**Interactive identification protocol.**
  *P* initialized with *x* communicates with *V* with *h*(*id*‖*i*) through the Stern identification protocol.

Cayrel et al. [8] show the mCFS-Stern scheme is id-imp-pa secure. Moreover, Yang et al. [21] proved the scheme also implies id-imp-aa secure. To achieve id-imp-ca secure, Yang et al. also proposed a new variant of the mCFS-Stern scheme, which introduced the OR-proof technique [7].

**Theorem 2.** *Yang’s identification protocol* (*P*, *V*) *is a proof of knowledge system with knowledge error* ${\left(\frac{2}{3}\right)}^{\gamma }$ [21].

**Remark.** *It should be noticed that the user key extraction of the mCFS-Stern scheme cannot resist the Bleichenbacher attack and the security proof relies on the indistinguishability between a binary Goppa code and a random code, which has been already invalidated*.

#### 2.2.4 Fiat-Shamir heuristic

According to Bellare et al. [6], identity-based signature (IBS) schemes could be constructed from convertible standard signatures or IBI schemes through Fiat and Shamir Heuristic. Unfortunately, code-based signature schemes, e.g., mCFS signature, are not convertible since no trapdoor samplable relation has been found to fit the key generation of existing signature schemes. Therefore, we adopt the latter method to construct IBS schemes.

Fiat and Shamir [24] proposed a general paradigm to drive a secure signature scheme from an identification scheme. Specifically, given a identification scheme with the commitment *α*, the challenge bit *β*, and the response *γ*, the signature for the message *m* is the transcript (*α*, *β*, *γ*), where *β* = *h*(*α*, *n*) and *h* is a cryptographic hash function. The verifier verifies the signature as *V* in the identification scheme. The paradigm will be used to derive the IBS schemes from our IBI schemes in the paper without security loss [25].

## 3 Provably secure IBI/IBS schemes

In this section, we propose a provably secure identity-based identification scheme, the PVR-Stern scheme. We first describe the scheme in Section 3.1, then we prove the scheme in Section 3.2.

### 3.1 Scheme description

The PVR-Stern scheme is id-imp-pa secure and we adopt the PVR signature technique in the user key extraction so that the security reduction is no longer depending on the indistinguishability between Goppa codes and random codes. We describe the scheme as follows:

**Master key generation.**
  1. Based on the input parameter 1^*κ*^, choose parameters *n*, *k*, $t=\frac{n-k}{\log2n}$, *n*′ = *n* − *k* + 1, and a cryptographic hash functions $G:F_{2}^{n-k}\times {\left\{0,1\right\}}^{n}\to F_{2}^{n^{\prime }}$.
  2. Select a (*n* − *k*) × *n* parity check matrix *H* of a *t*-error correcting binary Goppa code.
  3. Select a *n* × *n* permutation matrix *P*.
  4. Select a vector $a\overset{\$}{\leftarrow }F_{2}^{n^{\prime }}$.
  5. Select a vector $b\overset{\$}{\leftarrow }F_{2}^{n}$.
  6. Compute a (*n* − *k*) × *n*′ matrix *H*′ such that *H*′ *a*^*T*^ = 0.
  7. Select a full-rank matrix $Q^{\prime }\overset{\$}{\leftarrow }F_{2}^{n^{\prime }\times \left(n-k\right)}$, such that it makes a (*n* − *k*) × (*n* − *k*) matrix *Q* = *H*′ *Q*′ invertible.
  8. Generate a *n*′ × *n* parity check matrix $\tilde{H}=Q^{\prime }HP⊕a^{T}b$.
  9. If $\tilde{H}$ is not full-rank, choose another *b* to re-generate $\tilde{H}$ until it is full-rank.
  10. The master secret key *msk* = (*H*, *P*, *Q*, *H*′) and the master public parameters $mpk=(\tilde{H},n,k,t,n^{\prime },G)$.
**User key extraction.**
  1. Select $i\overset{\$}{\leftarrow }F_{2}^{n-k}$.
  2. Using the decoding algorithm to decode $Q^{-1}H^{\prime }G{\left(i,id\right)}^{T}$.
  3. If the decoding result *x*′ is not found, then go back to select *i* again.
  4. When *x*′ is found, *x* = *P*^*T*^*x*′, where wt(*x*) is *t* or less.
  5. The user public key is G(i,id), and the corresponding user secret key, *usk*[*id*] is *x*.
**Interactive identification protocol.**
  *P* initialized with *x* communicates with *V* with G(id∥i) through the Stern protocol.

### 3.2 Security

**Theorem 3.** *The PVR-Stern scheme is secure under passive attacks in the random oracle model*.

*Proof.* The proof adapts the reduction of the mCFS-Stern scheme [8] and PVR signature scheme [19]. It shows the advantage of an adversary A is equivalent to the advantage of breaking the BD problem through a series of games.

Let $q_{G},q_{E},q_{C}$ denote the maximum number of queries to hash oracle, user key extraction oracle and conversation oracle respectively. In each game, we maintain three lists $\Lambda_{G},\Lambda_{E},\Lambda$ to answer these queries. The list $\Lambda_{G}$ stores a tuple ((*s*, *x*), *a*) indexed by (*i*, *id*), where $i\overset{\$}{\leftarrow }F_{2}^{n-k}$, *id* is an identity and $\tilde{H}x^{T}=s=G\left(i,id\right)$. The list Λ_*E*_ stores usk[*id*] = (*i*, *x*) indexed by the identity *id*. The list Λ stores $i\overset{\$}{\leftarrow }F_{2}^{n-k}$ indexed by *m* ∈ {0, 1}*. ⊥ denotes the there is no value in the list.

**Game 0** is the standard id-imp-pa game. The master public and secret keys are obtained by the MKGen algorithm. The adversary A could issue initialization, conversation, or proving queries to the hash oracle and the user key extraction oracle. Let *X*_0_ be the event that A wins Game 0. Hence, Pr[*X*_0_] = ${\text{Adv}}_{A}^{\text{id}-\text{imp}-\text{pa}}\left(\kappa \right)$.

**Game 1** simulates the hash oracle for G and the user key extraction oracle.

The details of hash oracle simulation and user key extraction oracle simulation are given in Algorithm 1 and 2 respectively.

**Algorithm 1** Simulation of hash oracle.

**Input:** (*i*, *id*)

**Output:** A syndrome *s*

$\left(\left(s,x\right),x_{1}\right)\leftarrow \Lambda_{G}\left(i,id\right)$

 **if** *i* ≠ Λ(*id*) **then**

  **if** *s* = ⊥ **then**

$x_{1}\overset{\$}{\leftarrow }F_{2}^{n}$

$s\leftarrow \tilde{H}x_{1}^{T}$

   *x* ← ⊥

$\Lambda_{G}\left(i,id\right)\leftarrow \left(\left(s,x\right),x_{1}\right)$

  **end if**

  return G(i,id)=s

 **else**

  **if** *s* = ⊥ **then**

$x_{1}\overset{\$}{\leftarrow }F_{2}^{n}$ such that wt(*x*_1_ = *t*)

$s\leftarrow \tilde{H}x_{1}^{T}$

   *x* ← *x*_1_

$\Lambda_{G}\left(i,id\right)\leftarrow \left(\left(s,x\right),x_{1}\right)$

  **end if**

  return G(i,id)=s

 **end if**

If (*id*, *i*) is queried to hash oracle G and then Λ(*id*) is set to *i* randomly, the incoherence occurs and the user key extraction oracle aborts. Such event happens with the probability $\frac{q_{E}}{2^{n-k}}$. Let *X*_1_ be the event that A wins Game 1. Therefore, $|\mathrm{Pr}\left[X_{0}\right]-\mathrm{Pr}\left[X_{1}\right]|⩽\frac{q_{E}}{2^{n-k}}$.

**Game 2** changes user key extraction algorithm, it replaces *H* with *R* and $\tilde{H}$ with *R*′, where *R*′^*T*^ = [*R*^*T*^|*z*^*T*^], $R\overset{\$}{\leftarrow }F_{2}^{(n-k)\times n}$, and $z\overset{\$}{\leftarrow }F_{2}^{n}$. The adversary A can differentiate between Game 3 and Game 2 only if he can distinguish the random matrix *R*′ from $\tilde{H}$. Since *a*, *b*, *H*′ are secret and *b* cannot be identified from $\tilde{H}$ [19], such differentiation happens with negligible probability. Hence, *instead of depending on the probability to distinguish the Goppa code and the random code*, let *X*_2_ be the event that A wins Game 2, Pr[*X*_2_] = Pr[*X*_1_].

**Game 3** selects a random index $j\overset{\$}{\leftarrow }\left\{1,2,\cdots ,q_{G}+q_{E}+q_{C}\right\}$ as the target identity index. Select a syndrome $v\overset{\$}{\leftarrow }F_{2}^{n-k}$ and a random bit *v*_*b*_. We change the output syndrome of G to (*v*‖*v*_*b*_) when it comes to the *j*-th query by the adversary A. Let *X*_3_ be the event that A wins Game 3. The probability space is not modified since $\left(v∥v_{b}\right)\overset{\$}{\leftarrow }F_{2}^{n^{\prime }}$, therefore, Pr[*X*_3_] = Pr[*X*_2_].

**Game 4** modifies the winning condition so that if the impersonating identity is not equal to the target identity, then the game is aborted. Let *X*_4_ be the event that A wins Game 4. $\mathrm{Pr}\left[X_{4}\right]=\frac{\mathrm{Pr}[X_{3}]}{q_{G}+q_{E}+q_{C}}$.

**Game 5** answers conversation queries on the target identity in expected polynomial time according to [11]. Specifically, in each iteration of the identification protocol, it chooses one out of three cheating strategies randomly where each strategy succeeds with probability $\frac{2}{3}$. Let *X*_5_ be the event that A wins Game 5. The probability space is not modified and thus Pr[*X*_5_] = Pr[*X*_4_].

Based on Theorem 1, an adversary A impersonating the target identity with advantage ${\left(\frac{2}{3}\right)}^{\gamma }+\epsilon_{1}$ for a non-negligible *ϵ*_1_ > 0 can convert into a PPT algorithm solving the BD problem with probability $\frac{\epsilon_{1}^{3}}{10}$. Let C be the simulator for Game 5 using the input of the BD problem: ${\text{Adv}}_{C}^{\text{BD}}⩾\frac{{\left(\mathrm{Pr}\left[X_{5}\right]-{\left(\frac{2}{3}\right)}^{\gamma }\right)}^{3}}{10}$. Since ${\text{Adv}}_{A}^{\text{id}-\text{imp}-\text{pa}}\left(\kappa \right)=\mathrm{Pr}\left[X_{0}\right]⩾{\left(\frac{2}{3}\right)}^{\gamma }+\epsilon$, it can be calculated that $\epsilon ⩽\frac{q_{E}}{2^{n-k}}+10^{\frac{1}{3}}\left(q_{G}+q_{E}+q_{C}\right)\left({\left({\text{Adv}}_{C}^{\text{BD}}\right)}^{\frac{1}{3}}+\left(1-\frac{1}{\sqrt[{3}]{10}}\right){\left(\frac{2}{3}\right)}^{\gamma }\right)$. It means a successful adversary A implies a successful adversary against the BD problem. Therefore, the PVR-Stern scheme is id-imp-pa secure.

**Algorithm 2** Simulation of user key extraction oracle.

**Input:** *id*

**Output:** usk[id] = (*x*, *i*)

$i\overset{\$}{\leftarrow }F_{2}^{n-k}$

 Λ(*id*)←*i*

 run G(Λ(id),id)

$\left(\left(s,x\right),x_{1}\right)\leftarrow \Lambda_{G}\left(\Lambda \left(id\right),id\right)$

 **if** *x* = ⊥ **then**

  ABORT

 **else**

  Λ(*id*)←⊥

 **end if**

 return (*x*, *i*)

## 4 IBI/IBS schemes with parallel-PVR

In this section, we propose the parallel-PVR-caStern scheme. Compared to the PVR-Stern scheme, the parallel-PVR-caStern scheme is id-imp-ca secure and decreases the requirement of parameter choice for the same security level. We first describe the scheme in Section 4.1, then we discuss the security of the scheme in Section 4.2.

### 4.1 Scheme description

The parameter choice of the parallel-PVR-caStern scheme depends on the Bleichenbacher attack, which decreases the security level from $2^{\frac{mt}{2}}$ to $2^{\frac{mt}{3}}$, so we utilize the parallel-PVR signature technique to resist this attack. We convert the original counter-based PVR for the user key generation to complete decoding based PVR, so that we can construct parallel-PVR for better efficiency. Then we improve the security from id-imp-pa/aa secure to id-imp-ca secure through the OR-proof technique since the PVR-Stern scheme is id-imp-ca secure. We describe the scheme as follows:

**Master key generation.**
  The master key generation algorithm of parallel-PVR-caStern is identical to that of PVR-Stern except for some additional public parameters: cryptographic hash functions $G_{1},\cdots ,G_{\lambda }:{\left\{0,1\right\}}^{n}\to F_{2}^{n^{\prime }}$, injective mapping *ϕ*, parallel degree *λ* and additional weight *δ* for complete decoding such that $\left(\begin{array}{c} n\\ t+\delta \end{array}\right)>n^{t}$.
  The master secret key *msk* = (*H*, *P*, *Q*, *H*′) and the master public parameters $mpk=(\tilde{H},n,k,t,n^{\prime },\lambda ,G_{1},\cdots ,G_{\lambda },\phi ,\delta )$.
**User key extraction.**
  For *λ* signatures for the user identity *id* in parallel:
  1. Compute $s_{i}^{\prime }=G_{i}\left(id\right)$, where *i* ∈ {1, 2, ⋯, *λ*}.
  2. Compute $s_{i}=H^{\prime }s_{i}^{\prime T}$.
  3. Search all error patterns of *ϕ*_*δ*_(*j*) weight *δ*.
  4. Compute $s_{j,i}=s_{i}+\tilde{H}\phi_{\delta }{\left(j\right)}^{T}$
  5. Apply the decoding algorithm to the *s*_*j*, *i*_ where the result is *P*^*T*^ *Decode*_*H*_(*Q*^−1^*s*_*j*, *i*_).
  6. Once the decodable syndrome *s*_*j*_0_,*i*_ is found, then we have found a $p_{j_{0},i}^{\prime }$ such that $\tilde{H}\phi_{t}{\left(p_{j_{0},i}^{\prime }\right)}^{T}=s_{j_{0},i}$.
  7. The *i*th signature for the user identity *id* is $p_{j_{0},i}=\phi_{t+\delta }^{-1}\left(\phi_{t}\left(p_{j_{0},i}^{\prime }\right)+\phi_{\delta }\left(j\right)\right)$ such that $\tilde{H}\phi_{t+\delta }{\left(p_{j_{0},i}\right)}^{T}=G_{i}\left(id\right)$.
  8. The parallel signature for the user identity *id* is *x* = (*p*_*j*_0_,1_‖⋯‖*p*_*j*_0_,*λ*_).
  Run the above process twice to generate two different parallel signatures *x*_0_ and *x*_1_ for the user identity *id*, and toss a coin ϖ. The user public key is $\left(G_{1}\left(id\right)∥\cdots ∥G_{\lambda }\left(id\right)\right)$ and the corresponding user secret key *usk*[*id*] is $\left(\varpi ,x_{\varpi }\right)$.
**Interactive identification protocol.**
  For each *i* ∈ {1, 2, ⋯, *λ*}, the prover *P* is initialized with $\varpi ,p_{j_{0},i}\in x_{\varpi }$ to verify $\tilde{H}\phi_{t+\delta }{\left(p_{j_{0},i}\right)}^{T}=G_{i}\left(id\right)$, and the verifier *V* is initialized with the $G_{i}\left(id\right)$. The detail is as follows:
  **Commitment.**
    Based on $G_{i}\left(id\right)$ and *p*_*j*_0_,*i*_, calculate $c_{1}^{\varpi },c_{2}^{\varpi }$, and $c_{3}^{\varpi }$ according to the original Stern identification protocol. *P* randomly choose $b_{1-\varpi },b_{1-\varpi }^{\prime }\in \left\{0,1,2\right\}$. Based on the values of $b_{1-\varpi }$ and $b_{1-\varpi }^{\prime }$, select one of three impersonation strategies for Stern protocol listed follow and calculate corresponding $c_{1}^{1-\varpi },c_{2}^{1-\varpi }$, and $c_{3}^{1-\varpi }$:
    1. If $b_{1-\varpi }$ and $b_{1-\varpi }^{\prime }$ are not 0, change *y* in the original commitment to *y* ⊕ *ϕ*_*t*+*δ*_(*p*_*j*_0_,*i*_).
    2. If $b_{1-\varpi }$ and $b_{1-\varpi }^{\prime }$ are not 1, change *ϕ*_*t*+*δ*_(*p*_*j*_0_,*i*_) in the original commitment to a random vector *v* where wt(*v*) = *t*.
    3. If $b_{1-\varpi }$ and $b_{1-\varpi }^{\prime }$ are not 2, change *y* ⊕ *ϕ*_*t*+*δ*_(*p*_*j*_0_,*i*_) in the original commitment to *y* ⊕ *v* where $\tilde{H}v^{T}=G_{i}\left(id\right)$ and wt(*v*) is arbitrary.
    *P* sends $\left(c_{1}^{0},c_{2}^{0},c_{3}^{0},c_{1}^{1},c_{2}^{1},c_{3}^{1}\right)$ to *V*.
  **Challenge.**
    *V* randomly sends *b* ∈ {0, 1, 2} to *P*.
  **Answer.**
    1. *P* calculates $b_{\varpi }=b-b_{1-\varpi }\;\mathrm{mod}\;3$ and $b_{\varpi }^{\prime }=b-b_{1-\varpi }^{\prime }\;\mathrm{mod}\;3$.
    2. Based on $b_{\varpi }$ and $b_{\varpi }^{\prime }$, *P* calculates two responses $r_{\varpi }$ and $r_{\varpi }^{\prime }$ respectively according to the original Stern protocol.
    3. Based on $b_{1-\varpi }$ and $b_{1-\varpi }^{\prime }$, *P* calculates two responses $r_{1-\varpi }$ and $r_{1-\varpi }^{\prime }$ respectively according to the chosen impersonation strategy.
    4. *P* then sends $\left(b_{0},b_{1},b_{0}^{\prime },b_{1}^{\prime }\right)$ to *V*.
  **Check.**
    1. *V* checks whether $b_{0}\neq b_{0}^{\prime }$, $b_{1}\neq b_{1}^{\prime }$, $b_{0}+b_{1}=b\;\mathrm{mod}\;3$, and $b_{0}^{\prime }+b_{1}^{\prime }=b\;\mathrm{mod}\;3$.
    2. *V* then randomly sends *ρ* ∈ {0, 1} to *P*.
  **Response.**
    If *ρ* is 0, *P* sends *r*_0_ and *r*_1_.
    If *ρ* is 1, *P* sends $r_{0}^{\prime }$ and $r_{1}^{\prime }$.
  **Verification.**
    If *ρ* is 0, *V* checks *r*_0_ and *r*_1_.
    If *ρ* is 1, *P* checks $r_{0}^{\prime }$ and $r_{1}^{\prime }$.
  **Repeat.**
    Repeat the above four steps for *γ* times so that the expected security level is reached.

**Remark.** *In the practical implementation, the parity matrix* $\tilde{H}$ *may be hidden with the support and the generator polynomial of the Goppa code in the master key generation algorithm according to* [20, 26]. *Since the calculation of* $\tilde{H}$ *is a key point to avoid the assumption on the indistinguishability between Goppa codes and random codes, we still use original notions here for clarity*.

### 4.2 Security

We first consider the security of the PVR-caStern scheme, which could be regarded as a special case of the parallel-PVR-caStern scheme whose *λ* is always equal to one. Then we show the security of the parallel-PVR-caStern scheme.

**Theorem 4.** *The PVR-caStern scheme is secure against impersonation under active and concurrent attacks in the random oracle model*.

*Proof.* The proof is obtained by contradiction and adapting the proofs by the [7]. If there is an adversary A=(CV,CP) who can win the id-imp-ca game with non-negligible probability for the PVR-caStern protocol, then we can construct an adversary $F=(CV^{\prime },CP^{\prime })$ who can win the id-imp-pa game with non-negligible probability for the PVR-Stern protocol. The reduction from id-imp-ca secure to id-imp-pa secure shows below:

**Setup.**
  The security parameters *κ* and the master public key *mpk* are given to *CV*′.
**Learning Phase.**
  *CV*′ initializes *HU*, *CU*, *PS*, *USK*, where *USK* denotes the set of user secret keys. The security parameters *κ* and the master public key *mpk* are given to *CV* from *CV*′. *CV*′ simulates the oracles for *CV* as below.
  **Initialization.** If *id* ∈ *HU* ∪ *CU*, *CV*′ returns ⊥. Here *id* refers to the hash of the user identity as mentioned in the scheme. Otherwise, it sends (0, *id*) and (1, *id*) to the external initialization oracle. It tosses a coin $\varpi_{id}$ and sends it with the *id* to the external corruption oracle to obtain $usk\left[id\right]=\left(\varpi_{id},x_{\varpi_{id}}\right)$. Then it adds *id* and $\left(id,\varpi_{id},usk\left[id\right]\right)$ to *HU* and *USK* respectively. Finally, it tells *CV* whether the above process is successful.
  **Corruption.**
    If *id* ∉ *HU*, *CV*′ returns ⊥. Otherwise, *CV*′ removes *id* from *HU* and adds it into *CU*. It obtains $\left(id,\varpi_{id},usk\left[id\right]\right)$ from *USK* and returns *usk*[*id*] to *CV*.
  **Conversation.**
    If *id* ∉ *HU*, *CV*′ returns ⊥. Otherwise, *CV*′ sends (0, *id*) and (1, *id*) to the external conversation oracle to obtain the transcript $t=(c_{1}^{0},c_{2}^{0},c_{3}^{0},c_{1}^{1},c_{2}^{1},c_{3}^{1},b,b_{0},b_{1},b_{0}^{\prime },b_{1}^{\prime },\rho ,r_{0},r_{1},r_{0}^{\prime },r_{1}^{\prime })$. Then it returns *t* to *CV*.
  **Proving.**
    If *id* ∉ *HU*, *CV*′ returns ⊥. If (*id*, *s*) ∉ *PS*, then *CV*′ adds (*id*, *s*) to *PS*, picks a random bit *τ*, retrieves $\left(id,\varpi_{id},usk\left[id\right]\right)$ from *USK*, and sets a state of the prover *st*_*P*_[(*id*, *s*)] ← (*mpk*, *usk*[*id*], *τ*). Then *CV*′ computes *M*_*out*_ based on *M*_*in*_ in three cases: If *M*_*in*_ is a null string, *CV*′ sends $\left(id,1-\varpi_{id}\right)$ to the external conversation oracle to obtain the transcript. It extracts the three commitments $c_{1}^{1-\varpi_{id}},c_{2}^{1-\varpi_{id}},c_{3}^{1-\varpi_{id}}$ and set the remaining transcript to *st*_*P*_[(*id*, *s*)]. It then computes the commitments $c_{1}^{\varpi_{id}},c_{2}^{\varpi_{id}},c_{3}^{\varpi_{id}}$ with *id* and *usk*[*id*]. Then $M_{out}=\left(c_{1}^{0},c_{2}^{0},c_{3}^{0},c_{1}^{1},c_{2}^{1},c_{3}^{1}\right)$. If *M*_*in*_ is *b*, *CV*′ chooses $b_{1-\varpi_{id}}$ and $b_{1-\varpi_{id}}^{\prime }$ and computes the corresponding $b_{\varpi_{id}},b_{\varpi_{id}}^{\prime }$. $M_{out}=\left(b_{0},b_{1},b_{0}^{\prime },b_{1}^{\prime }\right)$. If *M*_*in*_ is *ρ*, *CV*′ computes responses $\left(r_{0},r_{1},r_{0}^{\prime },r_{1}^{\prime }\right)$ and set them to *M*_*out*_. Finally, *CV*′ returns *M*_*out*_.
**Challenge.**
  *CV* outputs a target identity id* and the state information *st*_*CP*_. If id* ∉ *HU*, then *CV*′ halts. Otherwise, *CV*′ gives *st*_*CP*_ to *CP*. Then *CV*′ acts as *V* to interact with *CP* multiple times so that transcripts of all the possible values of *b* and *ρ* are collected. With these transcripts, *CV*′ can compute the *usk*[id*]. *CV*′ outputs id* and corresponding $1-\varpi_{id^{*}}$ to challenger. After challenger returns (*mpk*, id*, *usk*[id*]) to *CP*′, *CP*′ acts as *P*′ to impersonate id* and $1-\varpi_{id^{*}}$.

F could impersonate $\left(1-\varpi_{id^{*}},id^{*}\right)$ successfully if ϖ is equal to $1-\varpi_{id^{*}}$ coincidently. Since A owns the user secret key *x*_0_ or *x*_1_ of *usk*[*id*] and the Reset Lemma [7, 27], ${\text{Adv}}_{F}^{id-imp-pa}\left(\kappa \right)⩾\frac{1}{2}{\left({\text{Adv}}_{A}^{id-imp-ca}\left(\kappa \right)-\frac{1}{∥G∥}\right)}^{2}$, where G is a commutative group over which the output challenge is uniformly distributed. Since ${\text{Adv}}_{F}^{id-imp-pa}\left(\kappa \right)$ is negligible according to Theorem 3, the PVR-caStern scheme is id-imp-ca secure.

**Theorem 5.** *The parallel-PVR-caStern scheme is secure against impersonation under active and concurrent attacks in the random oracle model*.

*Proof.* Based on Theorem 4, for each *i* ∈ {1, 2, ⋯, *λ*}, the *i*-th identification is secure under concurrent attacks in the random oracle model. Finiasz [20] has proposed that the parallel signatures keep a practical selection of parameters without the loss of security when the signing message (user identity here) is consistency, i.e., *λ* different cryptographic hashes for a user identity *id* constitute the user public key. Hence, since the PVR-caStern scheme is id-imp-ca secure, the parallel-PVR-caStern scheme is id-imp-ca secure.

## 5 Parameters and security

We compare the costs and sizes of the mCFS-Stern scheme and our four schemes as shown in Table 1. Our schemes differ in the ability to resist the Bleichenbacher attack (with/without parallel-PVR) and the security level (id-imp-pa/id-imp-ca). The mCFS-Stern scheme is not provably secure while our schemes are all provably secure.

**Table 1:** The asymptotic and estimate costs and sizes of our IBI/IBS schemes and the mCFS-Stern scheme.

Scheme	mpk Size	msk Size	usk Size	usk Cost	Communication Cost	Signature Length	Security
*mCFS-Stern*	*tm*2^*m*^	*tm*	*tm*	*t*!*t*^2^*m*^2^	2^*m*^*γ*	2^*m*^*γ*	Not Provably
	30MB	240	240	2^45^	2^26^	35MB	Secure
PVR-Stern	*tm*2^*m*^	*tm*	*tm*	*t*!*t*^2^*m*^3^	2^*m*^*γ*	2^*m*^*γ*	$2^{\frac{tm}{3}}$
	30MB	240	240	2^49^	2^26^	35MB	2^80^
PVR-caStern	*tm*2^*m*^	*tm*	*tm*	*t*!*t*^2^*m*^3^	2^*m*+1^*γ*	2^*m*+1^*γ*	$2^{\frac{tm}{3}}$
	30MB	240	240	2^49^	2^27^	70MB	2^80^
parallel-PVR-Stern	*tm*2^*m*^	*tm*	*λtm*	*λt*!*t*^2^*m*^3^	*λ*2^*m*^*γ*	*λ*2^*m*^*γ*	$2^{tm\frac{2^{\lambda }-1}{2^{\lambda +1}-1}}$
	5MB	162	324	2^38^	2^25^	18MB	2^77^
parallel-PVR-caStern	*tm*2^*m*^	*tm*	*λtm*	*λt*!*t*^2^*m*^3^	*λ*2^*m*+1^*γ*	*λ*2^*m*+1^*γ*	$2^{tm\frac{2^{\lambda }-1}{2^{\lambda +1}-1}}$
	5MB	162	324	2^38^	2^26^	35MB	2^77^

The mCFS-Stern scheme is the base scheme and our four schemes differ in the ability to resist the Bleichenbacher attack (with/without parallel-PVR) and the security level (id-imp-pa/id-imp-ca). For each scheme in the table, the upper row shows the asymptotic sizes and costs with the code length *m*, the error correcting capability *t*, the number of repetition *γ*, and the degree of parallelism *λ*. The lower row presents the estimated sizes (in bits) and costs (in the number of computations) with the parameters suggested by [8, 16, 19, 20].

### Parameters

For each scheme in the table, the upper row shows the asymptotic sizes and costs, and the lower row presents the estimated costs and sizes with the parameters suggested by [8, 16, 19, 20] to achieve a security level of about 2^80^. Specifically, for the schemes without parallel-PVR, *m* = log_2_ *n* = 20 and *t* = 12, otherwise, *m* = 18, *t* = 9, *λ* = 2, and *δ* = 2. For IBI schemes, the *γ* for communication cost is 58, and for converted IBS schemes through Fiat-Shamir paradigm, the *γ* for signature length is 280.

### Asymptotic analysis

The asymptotic sizes of parallel-PVR based schemes (*tm*2^*m*^ for *mpk* size, *tm* for *msk* size) are same with the schemes without Parallel-PVR technique. Also, parallel-PVR based schemes seem to cost more for their multiple signature and communication procedure. The asymptotic size of *usk* generation of parallel-PVR-Stern and parallel-PVR-caStern is *λtm*, which is *λ* times of PVR-Stern and PVR-caStern (*tm*). The situation is similar for the asymptotic cost of *usk* generation (*λt*!*t*^2^*m*^3^ and *t*!*t*^2^*m*^3^), communication cost (*λ*2^*m*^*γ* and 2^*m*+1^*γ*) and signature length (*λ*2^*m*^*γ* and 2^*m*^*γ*).

### Estimated costs and sizes

However, parallel-PVR based schemes actually decrease the parameters values, especially for *m* and *t* since the asymptotic security level is optimized from $2^{\frac{tm}{3}}$ to $2^{tm\frac{2^{\lambda }-1}{2^{\lambda +1}-1}}$. It shows that, with parallel-PVR, it improves a lot on *mpk* size (5MB and 30MB with/without parallel-PVR), *msk* size (162 bits and 240 bits), *usk* generation cost (2^38^ and 2^49^), communication cost (2^25^ and 2^26^ for id-imp-pa secure and 2^26^ and 2^27^ for id-imp-aa/ca secure) and signature length (18MB and 35MB for id-imp-pa secure and 35MB and 70MB for id-imp-aa/ca secure) with few costs of *usk* size (324 bits and 240bits). If id-imp-ca secure is required, the communication cost and signature length will be double compared to the lower security level.

As a result, with PVR, parallel-PVR and Or-proof techniques, it can be concluded that our schemes improve the efficiency of the mCFS-Stern scheme while maintaining the provable security. It represents an important advancement in the search for an ideal post-quantum identity-based identification and signature schemes.

## 6 Conclusion

In this paper, we propose identity-based identification and signature schemes from code assumptions with parallel-PVR. They are not only provably secure against impersonation under active and concurrent attacks but also have better efficiency.

It is worth noting that it still needs lots of works to study more robust assumptions on coding theory and construct broader identity-based cryptosystems from code assumptions. Also, we will make more efforts to achieve better system parameters so that code-based schemes will be more practical.

## Supporting information

S1 Table The asymptotic and estimate costs and sizes of our IBI/IBS schemes and the mCFS-Stern scheme. (PDF)

## References

[1] Shor PW. Algorithms for quantum computation: Discrete logarithms and factoring. In: 35th Annual Symposium on Foundations of Computer Science. IEEE; 1994. p. 124–134.

[2] McEliece R. A public-key cryptosystem based on algebraic. JPL DSN Progress Report. 1978;4244:114–116.

[3] Overbeck R, Sendrier N. Code-based cryptography. In: Proceedings of International Conference on Post-Quantum Cryptography. Springer; 2009. p. 95–145.

[4] Ezerman MF, Lee HT, Ling S, Nguyen K, Wang H. A Provably Secure Group Signature Scheme from Code-Based Assumptions. In: Proceedings of ASIACRYPT. Springer; 2015. p. 260–285.

[5] Shamir A. Identity-based cryptosystems and signature schemes. In: Proceedings of Workshop on the Theory and Application of Cryptographic Techniques. Springer; 1984. p. 47–53.

[6] Bellare M, Namprempre C, Neven G. Security proofs for identity-based identification and signature schemes. Journal of Cryptology. 2009;22(1):1–61. 10.1007/s00145-008-9028-8

[7] Fujioka A, Saito T, Xagawa K. Security enhancements by OR-proof in identity-based identification. In: Proceedings of International Conference on Applied Cryptography and Network Security. Springer; 2012. p. 135–152.

[8] Cayrel PL, Gaborit P, Galindo D, Girault M. Improved identity-based identification using correcting codes. CoRR, abs/09030069. 2009;.

[9] Courtois NT, Finiasz M, Sendrier N. How to achieve a McEliece-based digital signature scheme. In: Proceedings of ASIACRYPT. Springer; 2001. p. 157–174.

[10] Stern J. A new identification scheme based on syndrome decoding. In: Proceedings of CRYPTO. Springer; 1993. p. 13–21.

[11] Stern J. A new paradigm for public key identification. IEEE Transactions on Information Theory. 1996;42(6):1757–1768. 10.1109/18.556672

[12] Alaoui SMEY, Cayrel PL, Mohammed M. Improved identity-based identification and signature schemes using Quasi-Dyadic Goppa codes. In: Proceedings of International Conference on Information Security and Assurance. Springer; 2011. p. 146–155.

[13] Cayrel PL, Véron P, Alaoui SMEY. A zero-knowledge identification scheme based on the q-ary syndrome decoding problem. In: Proceedings of Conference on Selected Areas in Cryptography. Springer; 2010. p. 171–186.

[14] Aguilar C, Gaborit P, Schrek J. A new zero-knowledge code based identification scheme with reduced communication. In: Proceedings of IEEE Information Theory Workshop. IEEE; 2011. p. 648–652.

[15] Faugere JC, Gauthier-Umana V, Otmani A, Perret L, Tillich JP. A distinguisher for high-rate McEliece cryptosystems. IEEE Transactions on Information Theory. 2013;59(10):6830–6844. 10.1109/TIT.2013.2272036

[16] Finiasz M, Sendrier N. Security bounds for the design of code-based cryptosystems. In: Proceedings of ASIACRYPT. Springer; 2009. p. 88–105.

[17] Girault M, Cohen R, Campana M. A generalized birthday attack. In: Proceedings of Workshop on the Theory and Application of of Cryptographic Techniques. Springer; 1988. p. 129–156.

[18] Faugere JC, Otmani A, Perret L, De Portzamparc F, Tillich JP. Folding alternant and Goppa codes with non-trivial automorphism groups. IEEE Transactions on Information Theory. 2016;62(1):184–198. 10.1109/TIT.2015.2493539

[19] Preetha Mathew K, Vasant S, Rangan CP. On Provably Secure Code-Based Signature and Signcryption Scheme. IACR Cryptology ePrint Archive. 2012;2012:585.

[20] Finiasz M. Parallel-cfs. In: Proceedings of Conference on Selected Areas in Cryptography. Springer; 2010. p. 159–170.

[21] Yang G, Tan CH, Mu Y, Susilo W, Wong DS. Identity based identification from algebraic coding theory. Theoretical Computer Science. 2014;520:51–61. 10.1016/j.tcs.2013.09.008

[22] Berlekamp ER, McEliece RJ, Van Tilborg HC. On the inherent intractability of certain coding problems. IEEE Transactions on Information Theory. 1978;24(3):384–386. 10.1109/TIT.1978.1055873

[23] Dallot L. Towards a concrete security proof of Courtois, Finiasz and Sendrier signature scheme. Research in Cryptology. 2007; p. 65–77.

[24] Fiat A, Shamir A. How to prove yourself: Practical solutions to identification and signature problems. In: Proceedings of CRYPTO. Springer; 1986. p. 186–194.

[25] Pointcheval D, Stern J. Security proofs for signature schemes. In: Proceedings of EUROCRYPT. Springer; 1996. p. 387–398.

[26] Biswas B, Sendrier N. McEliece cryptosystem implementation: Theory and practice. In: Proceedings of International Conference on Post-Quantum Cryptography. Springer; 2008. p. 47–62.

[27] Feige U, Shamir A. Witness indistinguishable and witness hiding protocols. In: Proceedings of ACM Symposium on Theory of Computing. ACM; 1990. p. 416–426.

