# Supplementary material for: Provably secure identity-based identification and signature schemes from code assumptions
Source: PLoS One. 2017 Aug 15;12(8):e0182894. doi: 10.1371/journal.pone.0182894 (PMC5557532; doi:10.1371/journal.pone.0182894)
Supplement: S1 Table — (PDF) [file pone.0182894.s001.pdf]

Table 1. The asymptotic and estimate costs and sizes of our IBI/IBS schemes and the mCFS-Stern scheme.

Scheme	mpk Size	msk Size	usk Size	usk Cost	Communication Cost	Signature Length	Security
mCFS-Stern	$tm2^m$	tm	tm	$t!t^2m^2$	$2^m\gamma$	$2^m\gamma$	Not Provably Secure
	30MB	240	240	2^{45}	2^{26}	35MB	
PVR-Stern	$tm2^m$	tm	tm	$t!t^2m^3$	$2^m\gamma$	$2^m\gamma$	$2^{\frac{tm}{3}}$
	30MB	240	240	2^{49}	2^{26}	35MB	2^{80}
PVR-caStern	$tm2^m$	tm	tm	$t!t^2m^3$	$2^{m+1}\gamma$	$2^{m+1}\gamma$	$2^{\frac{tm}{3}}$
	30MB	240	240	2^{49}	2^{27}	70MB	2^{80}
parallel-PVR-Stern	$tm2^m$	tm	λtm	$\lambda t!t^2m^3$	$\lambda 2^m\gamma$	$\lambda 2^m\gamma$	$2^{tm\frac{2^\lambda-1}{2^{\lambda+1}-1}}$
	5MB	162	324	2^{38}	2^{25}	18MB	2^{77}
parallel-PVR-caStern	$tm2^m$	tm	λtm	$\lambda t!t^2m^3$	$\lambda 2^{m+1}\gamma$	$\lambda 2^{m+1}\gamma$	$2^{tm\frac{2^\lambda-1}{2^{\lambda+1}-1}}$
	5MB	162	324	2^{38}	2^{26}	35MB	2^{77}
